# A Novel High Throughput, Parallel Infection Assay for Determining the Replication Capacities of 346 Primary HIV-1 Isolates of the Zurich Primary HIV-1 Infection Study in Primary Cells

**DOI:** 10.3390/v13030404

**Published:** 2021-03-04

**Authors:** Audrey E. Rindler, Herbert Kuster, Kathrin Neumann, Christine Leemann, Dominique L. Braun, Karin J. Metzner, Huldrych F. Günthard

**Affiliations:** 1 Division of Infectious Diseases and Hospital Epidemiology, University Hospital Zürich, 8091 Zürich, Switzerland; audrey.rindler@gmail.com (A.E.R.); herbert.kuster@usz.ch (H.K.); kathrin.neumann@usz.ch (K.N.); christine.leemann@usz.ch (C.L.); dominique.braun@usz.ch (D.L.B.); 2 Institute of Medical Virology, University of Zürich, 8057 Zürich, Switzerland; 3 Life Sciences Graduate School, University of Zürich, 8057 Zürich, Switzerland

**Keywords:** HIV-1, replication capacity, primary cells, high throughput, parallel infection, primary HIV-1 isolates

## Abstract

HIV-1 replication capacity is an important characteristic to understand the replication competence of single variants or virus populations. It can further aid in the understanding of HIV-1 pathogenicity, disease progression, and drug resistance mutations. To effectively study RC, many assays have been established. However, there is still demand for a high throughput replication capacity assay using primary cells which is robust and reproducible. In this study, we established such an assay and validated it using 346 primary HIV-1 isolates from patients enrolled in the Zurich Primary HIV Infection study (ZPHI) and two control viruses, HIV-1 JR-CSF_WT_ and HIV-1 JR-CSF_K65R_M184V_. Replication capacity was determined by measuring the viral growth on PBMCs over 10 days by longitudinally transferring cell culture supernatant to TZM-bl reporter cells. By utilizing the TZM-bl luciferase reporter assay, we determined replication capacity by measuring viral infectivity. The simplicity of the experimental setup allowed for all 346 primary HIV-1 isolates to be replicated at one time. Although the infectious input dose for each virus was normalized, a broad range of replication capacity values over 4 logs was observed. The approach was confirmed by two repeated experiments and we demonstrated that the reproducibility of the replication capacity values is statistically comparable between the two separate experiments. In summary, these results endorse our high throughput replication capacity assay as reproducible and robust and can be utilized for large scale HIV-1 replication capacity experiments in primary cells.

## 1. Introduction

In order to understand viral behavior in regard to viral evolution [1,2], varying disease progression in the host (elite controllers, normal and rapid disease progression) [3,4,5,6,7,8], transmitted founder (T/F) virus characteristics [9,10,11,12,13,14], and fitness costs caused by drug resistance mutations or by positive and/or negative selection due to immune escape [15,16,17,18,19,20,21], it is important to study the replication capacities of HIV-1 variants. Over the years, different types of experimental assays have been designed and established to accommodate the specific questions that have been studied (reviewed in [22,23,24]). Unfortunately, it is uncommon for these assays to be high throughput due to the potential requirement for virus modifications and the various exhaustive quantification protocols used to determine the viral growth over time. It has been particularly difficult to establish large scale assays to determine RC in primary cells—the natural target cells for infectious viruses—because of the variability and availability of donor cells.

Viral replication capacity is determined by replicating the virus of interest (primary HIV-1 isolates, infectious molecular clones, pseudoviruses, chimeric viruses) on a specific cell type (primary cells or cell lines) and quantified using various established protocols (antigen ELISA, enzyme activity, flow cytometry, quantitative polymerase chain reaction (q-PCR), heteroduplex tracking assay (HTA)). Due to varying opinions, it is debated which assay is the best experimental structure for determining replication capacity. The two main setups that are discussed are dual competition infection and parallel infection.

Dual competition infection allows the viruses to be competed against one another in the same experimental environment, which is thought to mimic the in vivo competition a virus undergoes within the host [22,24]. This assay has the advantage that small differences can be observed between two viruses, but it also has disadvantages such that the quantification protocol is required to distinguish between the viruses, as well as the potential for recombination to take place. Many research groups tackle these limitations by making modifications to the viruses that can be distinguished using primers, Taqman probes or antibodies with fluorescent markers [5,25,26,27,28]. Furthermore, sequencing of the virus inoculum can help to determine if recombination occurred during the replication process [29]. Although there are ways to overcome the limitations associated with dual competition infection, modifications and sequencing are costly and time consuming which make it difficult to utilize for large sample sizes. On the other hand, the parallel infection assay setup allows each virus to be replicated in separate cell cultures.

Various quantification protocols can be used to determine the viral growth over time, but assays quantifying the amount of viral antigen (p24 ELISA) and enzyme activity (RT activity) are the most common [6,9,12,13,30]. More recently, protocols using TZM-bl cells that express luciferase under the control of the HIV-1 Tat promotor, have been used to determine viral infectivity, such as determination of the 50% tissue culture infectious dose (TCID_50_) [11,31,32]. Furthermore, parallel infection assays do not require modifications of the viruses and/or cells, which makes them cost effective and suitable for high throughput, large scale experiments.

There are established assays that can determine replication capacities of large sample populations of recombinant viruses that consist of a specific part of the patients’ viral genome (e.g., *gag*, *pol*) cloned into a control backbone (e.g., HIV-1 NL4-3) [30,31,32,33,34,35,36]. However, there is also need for an assay that can measure replication capacity of whole genome viruses in primary cells in a high throughput manner. A high throughput replication capacity assay can allow for all samples to be processed in one experiment while sufficiently producing large data sample sizes that can be utilized to draw robust statistical conclusions.

Therefore, the aim of this study was to establish a parallel infection, replication capacity assay for high throughput use in primary cells. The assay was validated using 346 primary HIV-1 isolates from the Zurich Primary HIV-1 Infection Study (ZPHI) and two control viruses, HIV-1 JR-CSF_WT_ and HIV-1 JR-CSF_K65R_M184V._ The replication capacities were determined by quantifying viral growth on primary peripheral blood mononuclear cells (PBMCs) over a period of 10 days using the TZM-bl luciferase assay and calculating the area under the curve (AUC) for the replication curves. The experimental assay was completed twice, and we compared those AUC values from the two experiments to determine the robustness and reproducibility of the assay. Furthermore, the AUC values were used to analyze the differences in replication capacities among primary HIV-1 isolates and assess the potential correlations between replication capacity and other viral characteristics. Taken together, these results show that we established a high throughput replication capacity assay based on primary cells that is reproducible and robust.

## 2. Materials and Methods

### 2.1. Patients

The ZPHI is an open label, non-randomized, observational, monocenter study at the University Hospital Zurich, Switzerland (www.clinicaltrials.gov, ID NCT00537966; approved by the ethical committee on September 2007 (KEK-ZH-Nr. EK-1452) [37,38,39]. All patients 18 years or older with documented acute or recent infection (see definition below), gave their informed consent and were included in the study. Patients with acute (<90 days) or recent (90–180 days) HIV-1 infection are enrolled) and antiretroviral therapy (ART) is immediately initiated among patients willing to start ART. The estimated date of HIV-1 infection (EDI) is determined based on unambiguous risk behavior, clinical (e.g., acute retroviral syndrome) and laboratory data (e.g., documented seroconversion, INNO-LIA, p24 antigen, Western Blot) [39].

### 2.2. Viruses

Primary HIV-1 isolates were derived using patients’ blood from the first visit during acute/recent HIV-1 infection. Briefly, CD4^+^ T cells and plasma were isolated from patients’ blood and co-cultured with 3-way stimulated, CD8-depleted, HIV-1 negative donor peripheral blood mononuclear cells (PBMCs) [40]. Primary HIV-1 isolates were characterized as follows: 50% tissue culture infectious dose (TCID_50_) was determined by serial diluting the viruses on TZM-bl cells [41]. Briefly, the viruses were cultured on TZM-bl cells for 3 days. TZM-bl cells were lysed, luciferase substrate (BrightGlo Luciferase Assay System, Promega, Madison, WI, USA) added, and the luciferase relative light units (RLU) was measured. According to the protocol, positive wells were defined by an RLU of at least twice the mean of the TZM-bl background controls [42].

For control viruses, the HIV-1 full-length plasmid pJR-CSF_WT_, was obtained through the NIH AIDS Reagent Program, Division of AIDS, NIAID, NIH: HIV-1 JR-CSF Infectious Molecular Clone (pYK-JRCSF) (Cat# 2708) from Dr. Irvin SY Chen and Dr. Yoshio Koyanagi [43,44,45]. The K65R and M184V mutations were introduced into the pJR-CSF_WT_ plasmid using the QuikChange XL Site-Directed Mutagenesis Kit (Agilent Technologies, Inc., Santa Clara, CA, USA) with sdm_M184V_fw (5′-CCAGACATAATTATCTATCAATACGTGGATGATTTGTATGTAGGATCTG-3′), sdm_M184V_rv (5′-CAGATCCTACATACAAATCATCCACGTATTGATAGATAATTATGTCTGG-3′), sdm_K65R_fw (5′-CTCCAGTATTTGCCATAAAGAGAAAAGACAGTACTAAATGGAG-3′) and sdm_K65R_rv (5′-CTCCATTTAGTACTGTCTTTTCTCTTTATGGCAAATACTGGAG-3′), following the manufacturer’s protocol. The primers were designed using the QuikChange Primer Design Tool offered by Agilent Technologies, Inc., accessed at https://www.agilent.com/store/primerDesignProgram.jsp [46] (accessed on 13 June 2018). The mutations were confirmed by next generation sequencing (NGS).

HEK293T cells were transfected with mixtures containing serum-free DMEM (no FBS, no PenStrep), 25 µg of plasmid (pJR-CSF_WT_ or pJR-CSF_K65R_M184V_) and 2 µg of polyethylenimine (PEI) per µg of DNA. After 48 h, the virus supernatants were harvested, filtered and the TCID_50_ was determined on TZM-bl cells as described above.

Real time quantitative polymerase chain reaction (RT-qPCR) was performed to determine the viral titer of the virus stocks as previously described [47]. Briefly, 150 µL of harvested virus stock was lysed and viral RNA was isolated (NucleoSpin™ RNA Virus, Macherey-Nagel™, Düren, Germany) according to the manufacturer’s protocol. Viral RNA was reverse transcribed into viral complementary DNA (cDNA) with primer RT_pol_3223 (5′-GGTTCTTTCTGATG-3′). The cDNA was amplified and quantified using primers pol_2981_fw (5′-TCAGTACAATGTGCTTCCACAGG-3′) and pol_3206_rc (5′-TTTGTCTGGTGTGGTAAATCCCCAC-3′) and SYBR green (Molecular Probes, Leiden, The Netherlands).

### 2.3. Comparing Parallel and Dual-Competition Infection

To evaluate parallel and dual competition infections, the viral titers were determined for HIV-1 JR-CSF_WT_ and HIV-1 JR-CSF_K65R_M184V_ and were used to calculate the amount of virus stock required for a multiplicity of infection (MOI) of 0.001. For the parallel infections, HIV-1 JR-CSF_WT_ and HIV-1 JR-CSF_K65R_M184V_, were added separately to tubes containing 2 × 10^6^ mixed donor, 3-way stimulated, CD8-depleted PBMCs. For the dual-competition infections, HIV-1 JR-CSF_WT_ was combined with HIV-1 JR-CSF_K65R_M184V_ at a 1:1 ratio for a combined MOI of 0.001 and added to 2 × 10^6^ mixed donor, 3-way stimulated, CD8-depleted PBMCs. The cells were infected by spinoculation for two hours at 1′200 g, washed with PBS, resuspended in RPMI 1640 with 10% heat inactivated FBS, 10 U/mL IL-2, and 1% P/S, and cultured on 12-well plates in duplicates [48]. On days 0 and 2–7 post infection (p.i), 50 µL and 150 µL of cell-free culture supernatant was extracted for p24 ELISA and RNA isolation, respectively. Each day cell-free culture supernatant was removed, the cells were replenished with 200 µL of RPMI 1640 medium with 10% heat inactivated FBS, 10 U/mL IL-2, and 1% P/S.

For both parallel and dual-competition infection data acquisition, p24 ELISA and RT-qPCR were performed. For p24 ELISA, 50 µl cell-free culture supernatant was inactivated with 1% Empigen and p24 antigen was quantified using an in-house p24 ELISA assay [49,50]. The RT-qPCR is described above. The viral copy numbers were calculated for each day and the replication growth kinetics was observed for parallel infection. In addition, for dual-competition infection data acquisition, the viral copy numbers of HIV-1 JR-CSF_WT_ and HIV-1 JR-CSF_K65R_M184V_ in the mixtures were separately measured with allele-specific polymerase chain reaction (AS-PCR). The validity of the K65R_M184V AS-PCR protocol was evaluated and confirmed as previously described [47,51]. AS-PCR was performed using primers IN_K65_fw (5′-TCCAGTATTTGCCATAAAGIA-3′) or IN_K65R_fw (5′-CCAGTATTTGCCATAAAGIG-3′), and pol_3206_rv (5′-TTTGTCTGGTGTGGTAAATCCCCAC-3′). Based on viral copy numbers of each variant, the replication capacity percentages were calculated to determine the competition kinetics of HIV-1 JR-CSF_WT_ and HIV-1 JR-CSF_K65R_M184V._

### 2.4. Cells

HEK293T cells were obtained through the NIH AIDS Reagent Program, Division of AIDS, NIAID, NIH: HEK-293 Cells from Dr. Andrew Rice [52]. The TZM-bl reporter cell line was obtained through the NIH AIDS Reagent Program, Division of AIDS, NIAID, NIH: TZM-bl cells (Cat#8129) from Dr. John C. Kappes, and Dr. Xiaoyun Wu [53,54,55,56]. The cells were cultivated in DMEM containing 10% heat inactivated fetal bovine serum (FBS) and 1% penicillin/streptomycin (P/S). When the TZM-bl cells were used for infection experiments, DMEM was supplemented with 10 µg/mL diethylaminoethyl-dextran (DEAE-Dextran) prior to infection.

Buffy coats from three HIV-1 negative donors were CD8-depleted (RosetteSep Human CD8 Depletion Cocktail, Stem Cell Technologies, Vancouver, British Columbia, Canada) and PBMCs were isolated by density gradient centrifugation. The cells were adjusted to 4 × 10^6^ per ml in RPMI 1640 containing 10% heat inactivated fetal bovine serum (FBS), 10 U/mL IL-2 and 1% penicillin streptomycin (P/S), split into 3 parts and stimulated with either 0.5 µg/mL phytohemagglutinin (PHA), 5.0 µg/mL PHA or OKT-3. After 48 h, the three stimulated parts were combined and cultured in RPMI 1640 with 10% heat inactivated FBS, 50 U/mL IL-2 or 10 U/mL IL-2, and 1% P/S [40].

### 2.5. High-Throughput Parallel Replication Capacity Assay

**Figure 1:**
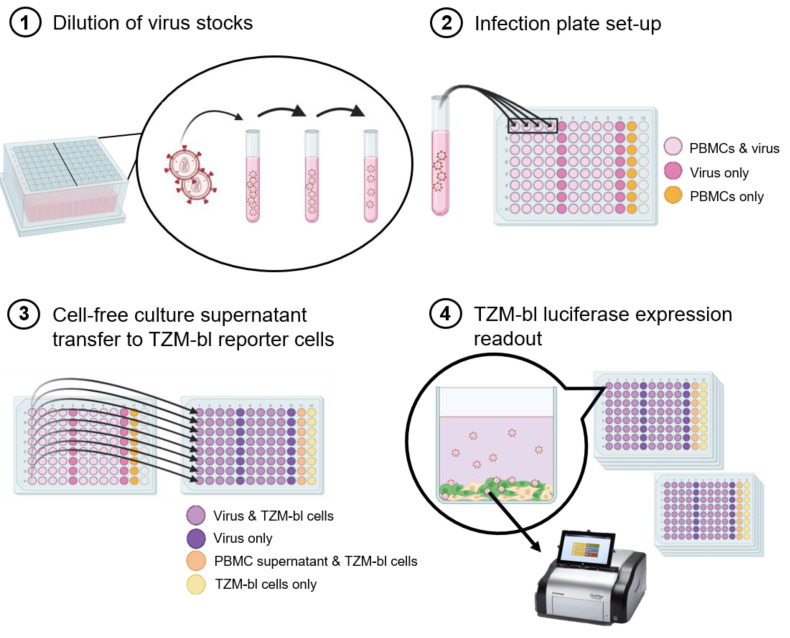
Workflow schematic of the high-throughput parallel replication capacity assay. (**1**) The virus stocks were added to the dilution tubes and diluted 1–5 times, depending on the TCID_50_/mL of the stock, in order to reach 2000 TCID_50_/mL. A total of 16 viruses could be diluted in one dilution box. (**2**) In quadruplicates, 100 μL of diluted virus was added to wells with 2 × 10^5^ PBMCs (light pink wells) and to wells with only RPMI medium (dark pink wells). In addition, a row of 8 wells containing PBMCs only (no virus) was added as a control (orange wells). (**3**) Starting three days post infection, 10 μL of cell-free supernatant was transferred from the infection plates to plates containing 2 × 10^4^ TZM-bl reporter cells per well (light purple wells: virus and TZM-bl reporter cells; dark purple wells: virus only; light orange wells: PBMC supernatant and TZM-bl reporter cells; light yellow wells: TZM-bl reporter cells only) at days 3, 4, 5, 6, and 7, post infection. The transfer was made each day starting at the same hour every day. (**4**) After 24 h incubation (starting at the same hour everyday), in batches of 4 plates per batch, the TZM-bl cells were lysed and the luciferase expression was measured.

The workflow is shown in Figure 1. Briefly, to determine the replication capacities of 346 primary HIV-1 isolates, they were serially diluted in cell culture medium to 2000 TCID_50_/mL (Figure 1.1). Notably, a new frozen vial of virus stock was utilized for each experiment. Therefore, there were no freeze/thaw cycles, as the aliquots were utilized on a one-time basis. In quadruplicates, 100 µL virus dilution was added to 96-well plates (Eppendorf 96-well cell culture plate) which contained 2 × 10^5^ stimulated CD8-depleted PBMCs in 100 µL cell culture medium (Figure 1.2). On days 3, 4, 5, 6, and 7 post infection (p.i.), at the same hour of the day, 10 µL of cell-free culture supernatant was transferred onto TZM-bl luciferase reporter cells (Figure 1.3). The TZM-bl luciferase expression (relative light units, RLU) was measured 24 h after the harvest transfer for each day in batches (Figure 1.4). All manipulations were performed in timed batches of four plates to minimize variability of incubation times throughout the entire assay.

Twice the mean of the PBMC background control on each plate was subtracted from each RLU value of each virus on the same plate. Next, the replication capacity of each virus was determined by calculating the area under the median replication curve (AUC) of the quadruplicates. This resulted in one replication capacity value for each virus.

### 2.6. Statistical Analysis

The statistical analysis was performed using both Prism and R Studio. Comparisons between two variables were performed using the Wilcoxon matched pairs signed rank test. If more than two variables were compared, the Kruskal–Wallis test was performed. The correlations were determined using linear regression analysis. *p*-value ≤ 0.05 was considered statistically significant.

## 3. Results

### 3.1. Characterization of Control Viruses

To achieve control viruses that represent both high and low replication competence, the HIV-1 full-length wildtype plasmid pJR-CSF_WT_ was used to produce the high replication competent virus, as well as, utilized to generate the less replication competent virus, pJR-CSF_K65R_M184V_. M184V and K65R are drug resistance mutations located in the reverse transcriptase of the viral *pol* gene. It has been previously shown that the K65R mutation reduces replication capacity up to 55% compared to the wildtype virus, but when paired with the M184V mutation replication capacity was reduced up to 70% compared to the wildtype in in vitro experiments [57]. The two mutations were introduced into the wildtype plasmid using site-directed mutagenesis. NGS analysis confirmed the absence of both mutations in pJR-CSF_WT_, while pJR-CSF_K65R_M184V_ contained both mutations. Virus stocks were generated by transfection of HEK293T cells and harvesting filtered cell-free culture supernatant 48 h post transfection.

The replication capacities of the control viruses were characterized by infecting pooled three donor, 3-way stimulated, CD8 depleted PBMCs and acquiring cell-free culture supernatant at days 2–7 (Figure S1A,B). The parallel infection replication curves depict a clear difference with HIV-1 JR-CSF_WT_ replicating more efficient than HIV-1 JR-CSF_K65R_M184V_ (Figure S1A). Furthermore, we performed a dual-competition experiment and determined the percentage of each viral variant in the mixture. The mixture ratio at day 2 was approximately 50/50, but over the course of the experiment, HIV-1 JR-CSF_WT_ replicated at a higher efficiency and outcompeted HIV-1 JR-CSF_K65R_M184V_ (Figure S1B)_._

### 3.2. Assay Design and Validation

To determine the optimal assay conditions for the high-throughput replication capacity assay based on primary cells, we performed a series of experiments with varying conditions using primary HIV-1 isolates from 14 patients and the two control viruses. The primary HIV-1 isolates were chosen based on the log TCID_50_/mL of the virus stock and ranged from 3.5–7.6 log TCID_50_/mL. All experiments were performed by parallel infection of pooled three donor, 3-way stimulated, CD8 depleted PBMCs with cell-free culture supernatant samples taken at days 3, 4, 5, 6, 7 and day 10.

**Figure 2:**
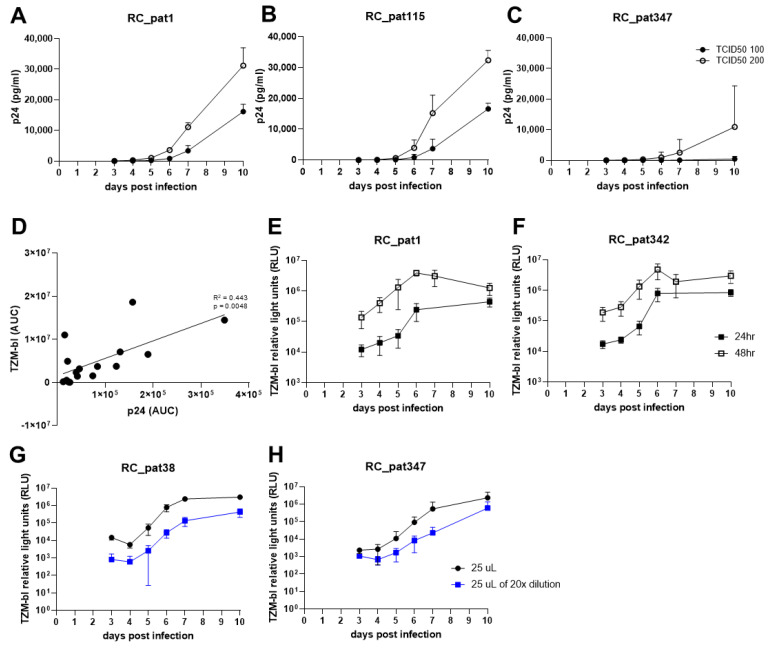
The optimization experiments for establishment of the replication capacity assay. (**A**–**C**) The graphs depict the replication kinetics comparison between infection with TCID_50_ 100 and 200 for three primary HIV-1 isolates. The x-axis represents the days post infection, and the y-axis represents p24 antigen value (pg/mL) measured by p24 ELISA in linear-scale. (**D**) The scatterplot compares AUC values obtained from two separate quantifying methods. The x-axis represents the AUC values obtained by quantifying p24 antigen. The y-axis represents the AUC values obtained from quantifying TZM-bl luciferase expression. An OLS regression line is shown (black line). Both axes are in linear-scale. (**E**,**F**) The graphs show TZM-bl luciferase expression measured 24 h (closed black square) or 48 h (open black square) after culture supernatant transfer to TZM-bl cells. The x-axis represents the days post infection, and the y-axis represents the TZM-bl luciferase expression measured by relative light units (RLU). (**G**,**H**) The graphs depict the comparison of different viral input amounts onto TZM-bl cells in log-scale. The x-axis represents the days post infection, and the y-axis represents the TZM-bl luciferase expression measured by relative light units (RLU) in log-scale.

First, we assessed the amount of infectious virus required for multiple rounds of infection. We tested both 100 and 200 TCID_50_/well of infectious virus input and determined the replication capacity of each virus by quantifying the p24 antigen production and evaluating the replication curves gained over the period of 10 days (Figure 2). The replication kinetics for 100 and 200 TCID_50_/well were similar (Figure 2A,B). However, for the primary HIV-1 isolates that depicted to be less replication competent, 100 TCID_50_/well was an inadequate amount of infectious virus to allow for efficient replication (Figure 2C). On the contrary, for the same viruses, 200 TCID_50_/well exhibited proficient viral replication (Figure 2C). Therefore, 200 TCID_50_/well was used for all further RC experiments.

Second, we examined whether viral replication should be quantified using either p24 ELISA or TZM-bl luciferase expression due to the requirement for the final assay to be high-throughput. To assess the similarities of the two quantification assays, cell-free culture supernatant was taken at days 3, 4, 5, 6, 7 and 10 and then split for quantification using either p24 ELISA or the TZM-bl reporter assay. The area under the curve (AUC) was calculated for the replication curves of the input of 200 TCID_50_/well infectious virus for the 14 primary HIV-1 isolates and two control viruses. A linear regression analysis was performed using the AUC values for both p24 antigen and TZM-bl luciferase expression corresponding to each virus (Figure 2D). The comparison shows that the two assays are comparable (R^2^ = 0.433, *p* = 0.005), which indicates that the HIV-1 p24 antigen produced during replication is proportional to the amount of infectious virus. The TZM-bl luciferase reporter gene assay can be easily scaled up for processing more samples, when compared to p24 ELISA. Furthermore, it measures viral infectivity. Therefore, the TZM-bl assay was used for all further experiments.

Third, we optimized the TZM-bl assay regarding the virus input on the TZM-bl cells. First, we assessed the incubation time regarding viral replication on the TZM-bl cells. For this experiment, cell-free culture supernatant was transferred from the plate containing HIV-1 infected PBMCs to the TZM-bl cells on days 3, 4, 5, 6, 7 and 10, and the luciferase expression was measured after 24 h and 48 h post infection. The replication curves for 48-h read out were approximately 1-log higher when compared to the 24-h replication curves (Figure 2E,F). This indicates that the viruses were undergoing multiple rounds of infection during the 48-h period leading to exhaustion of the TZM-bl cells and limiting the dynamic range of the reporter assay. Since we aim to quantify the virus produced during replication on the primary cells, one round of replication on the reporter cell line is favored over multiple rounds in order to acquire a more exact quantification of both replication capacity and viral infectivity. Therefore, quantification of TZM-bl luciferase expression after 24 h was preferred over 48 h.

Forth, we aimed to determine the optimal amount of virus to transfer to the TZM-bl cells. We transferred either 25 µL of undiluted or 25 µL of 1:20 diluted cell-free culture supernatant to TZM-bl cells. By lowering the cell-free culture supernatant input by 50%, the luciferase expression values stayed relatively the same when compared to 100% input (50 µL) at 10^4^–10^5^ relative light units (RLU). Furthermore, the lowest input of 25 µL of 1:20 dilution (equal to approximately 1.25 µL of original infection mixture) depicted a decrease of luciferase expression at day 3 by at most 1-log (10^3^–10^4^ RLU) (Figure 2G,H). These results indicate that by decreasing the cell-free culture supernatant input onto TZM-bl, it does lower the luciferase expression at day 3 but only minimally.

**Figure 3:**
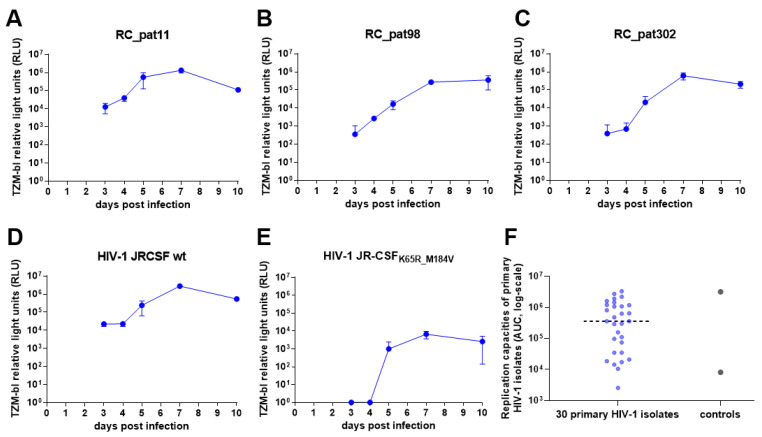
Pilot experiment of the optimized high throughput parallel infection assay using 30 primary HIV-1 isolates. (**A**–**E**) These graphs depict the replication kinetics from three primary HIV-1 isolates (**A**–**C**) and the two control viruses (**D**,**E**). The x-axes represent the days post infection, and the y-axes display the TZM-bl luciferase expression measured by relative light units (RLU) in log-scale. (**F**) The jitter plot depicts the distribution of the replication capacities for the 30 primary HIV-1 isolates and the control viruses. The x-axis depicts the samples, and the y-axis depicts the replication capacities (AUC values) in log-scale. The dotted horizontal line represents the median.

Following these optimization steps, the replication capacity assay was performed using 30 patient HIV-1 primary isolates to test the experimental set-up and optimized conditions of the assay (Figure 3). The replication kinetics display exponential growth over time with a plateau occurring around day 7 (Figure 3A–E). Furthermore, HIV-1 JR-CSF_WT_ replicates at a higher efficiency when compared to the replication kinetics of HIV-1 JR-CSF_K65R_M184V_ (Figure 3D,E). Although the replication kinetics were as expected, from day 7 to day 10, the majority of the RLU values either stayed the same or dropped, which suggests that saturation of virus occurs sometime between day 7 and 10. Therefore, day 10 was excluded from the AUC calculation and from all further experiments. Of note, we evaluated different ways to characterize the replication curve, like slope, RLU at day 7 and AUC. We determined that AUC is more robust by considering the data points from the entire experiment rather than utilizing only a few data points. The replication capacities of the primary HIV-1 isolates exhibited a large distribution from 10^3^–10^6^, with the two control viruses representing the high (HIV-1 JR-CSF_WT_) and low (HIV-1 JR-CSF_K65R_M184V_) replication capacities (Figure 3F). Interestingly, one primary HIV-1 isolate had a lower replication capacity than HIV-1 JR-CSF_K65R_M184V_.

### 3.3. Determining the Replication Capacities of 346 Primary HIV-1 Isolates Using the Optimized High-Throughput Parallel Infection Assay

Using the optimized high throughput parallel replication capacity assay, we determined the replication capacities of 346 primary HIV-1 isolates in a single experiment in primary cells.

Twenty-seven 96-well infection plates had 16 viruses in quadruplicates with individual virus input controls for each virus, as well as a column of uninfected PBMCs (Figure 1.2). The Eppendorf 96-well plates contained a moat around the outside of the wells and room between wells to allow the outer and entire inner-well area to be filled with approximately 4 mL of PBS, allowing for the use of all 96 wells without risk of evaporation for up to 10 days. On every fourth infection plate, the two control viruses, HIV-1 JR-CSF_WT_ and HIV-1 JR-CSF_K65R_M184V_, were added as intra-assay controls. To avoid potential bias due to long time storage, all virus stocks were titrated on TZM-bl cells within twelve weeks, with the log TCID_50_ values ranging from 3.45 to 8.16. The viruses were then organized by the number of dilution steps needed to attain the required infectious dose of 2000 TCID_50_/mL. Dilution boxes were organized with 16 viruses for each box and labeled with numbers that corresponded to the number on the infection plate (Figure 1.1). This protocol allowed a high-throughput dilution and infection process of more than 400 virus samples in total. Each day at the same hour, starting three days post infection, 10 μL of cell-free culture supernatant from the infection plates was transferred to 2 × 10^4^ freshly passaged TZM-bl cells on the reporter plates (Figure 1.3). After 24 h incubation, the luciferase expression was quantified in batches (4 plates at a time) (Figure 1.4). By using a batch-dependent routine, the potential for luciferase expression degradation over time was minimized. That routine was repeated over the period of 7 days. Before determining the replication capacity, the background must be subtracted from each virus RLU value to control for the baseline luciferase expression. According to the standard TZM-bl luciferase assay protocol, the mean of the TZM-bl background control should be subtracted from the virus

RLU value [42]. However, we found that the PBMC supernatant on TZM-bl cells (PBMC background control) values were higher than the TZM-bl background control (Figure S2). This indicated that potential unknown factors in the IL-2 containing RPMI medium could be increasing the baseline luciferase expression. Therefore, to control for the increase in baseline luciferase expression, we subtracted the mean of the PBMC background control value. Next, the median area under the curve (AUC) was calculated for the quadruplicates of each primary HIV-1 isolate. Therefore, each replication capacity is denoted by one median AUC value per primary HIV-1 isolate.

**Figure 4:**
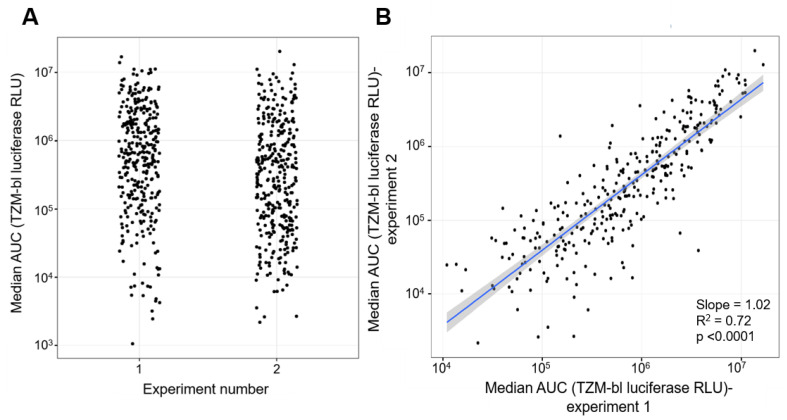
Comparison of replication capacities of 346 primary HIV-1 isolate obtained from two separate experiments. (**A**) Distribution of the replication capacities (AUC) of 346 primary HIV-1 isolates for each experiment. The x-axis denotes the experiment from which the AUC values were measured. The y-axis depicts the replication capacities of the primary HIV-1 isolates represented as AUC values. (**B**) Scatterplot comparison of AUC values obtained from two separate experiments. The x-axis represents the values for the first experiment and the y-axis represent the values for the second experiment. Both axes are in log scale. An OLS regression line is shown (blue line) with its standard error band (gray shaded area).

To test the reproducibility, the large-scale replication capacity assay was performed twice. The replication kinetics of the control viruses were similar for both experiments (Figure S3A,C). HIV-1 JR-CSF_K65R_M184V_ replicated more than 3-logs lower than HIV-1 JR-CSF_WT_. The distributions of the replication capacities of the primary HIV-1 isolates were broad, ranging from 10^3^ to 10^7^ median AUC, for both experiments (Figure 4A). In addition, all replication capacities of the primary HIV-1 isolates were between the average replication capacities of the two control viruses. The scatter plot of the median AUC values is shown in Figure 4B. A standard regression analysis was performed, and the slope of the line is 1.02 (*p*-value < 0.0001) with a R^2^ of 0.72. This shows that for each virus, the replication capacity is highly reproducible in repeated experiments. Given this result, all the following analyses are based on the median AUC values obtained in the second experiment.

### 3.4. Comparisons of Replication Capacities to Viral Characteristics of Primary HIV-1 Isolates

Characteristic summary of the primary HIV-1 isolates used in the experiments are shown in Table 1. The samples were obtained over a period of 15 years, from year 2002 through 2017. Most of the primary HIV-1 isolates were obtained during the acute stage (<90 days) of HIV-1 infection (*n* = 265, 76.6%). The primary HIV-1 isolates were predominantly derived from patient CD4^+^ T cells (*n* = 322, 93%). During derivation of the primary HIV-1 isolates, the average number of days in culture was 20 days with the minimum and maximum at 5 and 48 days, respectively. The average titer of the primary HIV-1 isolate stocks was 5.28 logTCID_50_/mL (min = 3.45, max = 8.16). The majority of primary HIV-1 isolates are HIV-1 subtype B (*n* = 256, 70%). The circulating recombinant HIV-1 forms (CRF) make up 13.4% (*n* = 46) of primary HIV-1 isolates subtypes with CRF01_AE and CRF02_AG being the most dominant at 8.1% and 2.9%, respectively.

**Figure 5:**
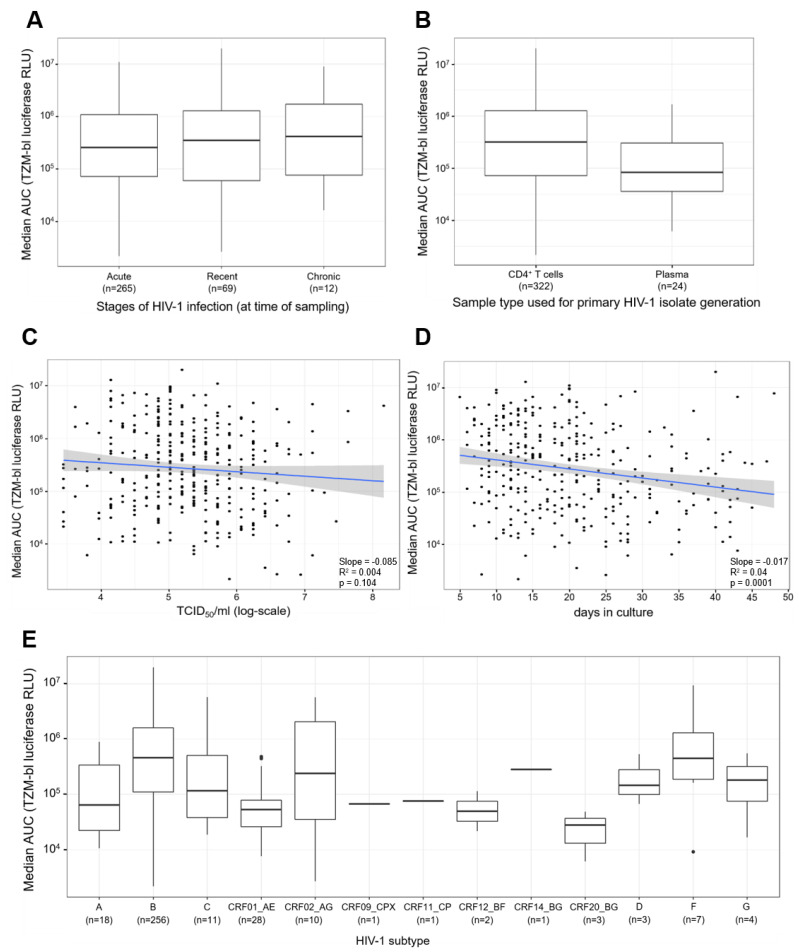
Comparisons of primary HIV-1 isolate replication capacities to stages of infection, types of samples used for generation of primary HIV-1 isolates, viral titers of virus stocks, days in culture, and HIV-1 subtypes. (**A**) Comparison of replication capacities of primary HIV-1 isolates for the different stages of HIV-1 infection. The x-axis represents the three stages of infection: acute, recent, and chronic, and the number (n) of primary HIV-1 isolates in each stage of infection. The y-axis represents the median AUC values of the replication capacities in log scale. The Wilcoxon signed-rank test was performed for comparisons between two groups and the Kruskal–Wallis test was performed for comparisons of more than two groups. (**B**) Comparison of the replication capacities of primary HIV-1 isolates derived from CD4^+^ T cells or plasma. The x-axis depicts the 2 types of samples used to generate the primary HIV-1 isolates, and the number (n) of primary HIV-1 isolates derived from the corresponding sample type. The y-axis represents the median AUC values of the replication capacities of the primary HIV-1 isolates. (**C**) Comparison of the viral titers of the primary HIV-1 isolate stocks to the corresponding replication capacities. The x-axis represents the tissue culture infectious dose of 50% per ml in log-scale. The y-axis depicts the median AUC values of the replication capacities for the primary HIV-1 isolates. (**D**) Comparison of the number of days the T/F primary isolates were in culture on the x-axis against the median AUC values of the replication capacity of the viruses on the y-axis, in log scale. The graph shows the OLS regression results (blue line) and its standard error (gray shading). (**E**) Comparison of the distribution of the replication capacity for different HIV-1 subtypes. The x-axis represents the various HIV-1 subtypes and the number (n) of each subtype. The y-axis depicts the replication capacities of T/F viruses represented as median AUC values. The black dots represent outliers, identified as values that are more than 1.5 times the interquartile range removed from the first or third quartile. The Kruskal Wallis test was performed to compare the median AUC values among the different HIV-1 subtypes.

Next, we compared the distribution of the replication capacities for the different stages of the HIV-1 infection at the time of sampling (Figure 5A). The comparison shows that the distribution of the median AUC values for the three stages are broadly the same, with a mild increase in the central values observable for chronic patients. The Kruskal–Wallis test was performed to determine the differences in mean values of the median log-AUC values and the comparisons showed that the differences between the three groups are statistically not significant (*p*-value = 0.763). In addition, all three pair-wise comparisons similarly show that the differences are statistically not significant (acute-chronic *p*-value = 0.552, acute-recent *p*-value = 0.637, recent-chronic *p*-value = 0.685).

Furthermore, we compared the distribution of the replication capacities for the two different sample types that were utilized to generate the primary HIV-1 isolates (Figure 5B). The comparison revealed the median AUC values for primary HIV-1 isolates derived from CD4^+^ T cells were higher compared to the median AUC values for plasma derived primary HIV-1 isolates. Furthermore, a Wilcoxon rank sum test confirmed that the differences between CD4^+^ T cells and plasma are statistically significant (*p*-value = 0.007).

In addition, we compared both the viral titer of the virus stocks (TCID_50_/_mL_) and the number of days the patient CD4^+^ T cells or plasma was co-cultured with PBMCs when deriving the primary HIV-1 isolates, against the median AUC values of the replication capacity of the primary HIV-1 isolates (Figure 5C,D). For the viral titers of the virus stocks, we observed a negative slope of −0.085 with a *p*-value of 0.104 and a R^2^ of the regression of 0.04 (Figure 5C). This result indicates that there was no association between viral titer of the virus stocks and the replication capacities. In contrast, the comparison between the number of days in culture to the replication capacities revealed a negative slope of −0.0174 with a *p*-value of 0.0001 and a R^2^ of the regression of 0.044 (Figure 5D). This result indicates that the primary HIV-1 isolates with higher replication capacities tend to require a reduced amount of time until harvest compared to lower replicating viruses.

Finally, we compared the distribution of 13 different HIV-1 subtypes (Figure 5E). The boxplots demonstrate that various primary HIV-1 isolate subtypes exhibit differing levels of replication capacity. The differences in central tendencies are statistically significant as confirmed by a Kruskal–Wallis test (*p*-value < 0.0001). It is notable that HIV-1 subtype B presents a high replication capacity, as does HIV-1 subtype F.

## 4. Discussion

In this study, we established a high-throughput parallel replication capacity assay using primary cells to study the replication capacities of 346 primary HIV-1 isolates of the Zurich Primary HIV-1 Infection Study. Although the assay was established to study primary HIV-1 isolates, we show that our assay is versatile and can be utilized to determine the HIV-1 replication capacity regarding many other topics such as fitness costs due to drug resistance mutations.

It was important for the assay to determine the replication capacity using the whole virus because of the potential influence that multiple protein interactions could have on replication capacity [58]. Furthermore, it has been found that regions other than Env, such as Pol and Gag, could contribute to replication capacity and fitness [22]. Due to the necessity to quantify the amount of viral growth over time, especially when performing dual competition infections, modifying the genome is common to distinguish between the different viruses. However, modifications can be time consuming, costly, and sometimes difficult. Although growth competition/dual competition assays are considered the gold-standard method for determining viral replication capacity and/or fitness [22,24], we found that parallel infection was non-inferior to dual competition infection. By using the two control viruses that represent high and low replication competent viruses, we saw that the differences between replication kinetics obtained from parallel infection were clearly observed for both viruses. In addition, dual competition assays only allow the comparison of one viral variant to another. It would have been near to impossible to make all the necessary comparisons among 346 viruses. We were able to overcome the argument that dual competition is superior because it allows for the viruses to grow in the same conditions, by replicating and comparing all 346 viruses at one time in separate wells while using the same primary cells and culture medium for all viruses. Furthermore, recombination was not possible due to infection with a low MOI of 0.0007 and by employing parallel infection [22].

For this high-throughput parallel infection assay, we infected primary cells. It can be argued that using primary cells for infection assays from multiple healthy donors can increase the likelihood of donor variability [11,59,60]. However, by combining PBMCs from three donors and stimulating the cells three ways, we were able to reduce the intra-patient variability regarding the susceptibility of HIV-1 infection [40]. We demonstrate that the reproducibility of the replication capacity values is statistically comparable between two separate experiments using PBMCs from a total of six different donors (three donors combined per experiment).

There are many different methods for quantifying HIV-1 viral growth over time. In this study, we compared the p24 ELISA method to the TZM-bl luciferase expression method and a correlation was observed between the median AUC values obtained from each assay. Our findings coincide with Etemad et al., 2014, who previously found that p24 antigen measurements were highly correlated with TZM-bl infectivity values, suggesting that either method could be used to quantify viral growth [11]. By using the TZM-bl luciferase expression method, we were able to measure the infectivity of the viruses. This is the reason that the viruses are first replicated on PBMCs (initial inoculum) and then cell-free culture supernatant is transferred to the TZM-bl cells, which then infect and undergo one round of replication, which has been found to occur after approximately 24 h [61]. Therefore, the TZM-bl luciferase expression that is quantified by luminescence is directly proportional to the number of infectious viral particles that are present in the initial inoculum [42].

Although quite a lot has been published about primary HIV-1 isolate replication capacities, the labor-intensive nature of the replication assays produces limitations regarding the sample sizes of the studies that compare replication fitness to viral characteristics [22]. By using the high throughput parallel replication capacity assay described in this paper, we were able to compare the replication capacities of 346 primary HIV-1 isolates and observe a broad range of median AUC values (10^3^–10^7^) when the infectious input dose for each virus was normalized to 200 TCID_50_/well. Furthermore, we saw no significant differences among the replication capacities of primary HIV-1 isolates classified by their infection stage of acute, recent, or chronic. Other researchers have found similar results regarding replication capacities among primary HIV-1 isolates obtained in different infection stages [9,11,12,31], while others have found significant differences among replication capacities of primary HIV-1 isolates compared to other isolates obtained from the donor [13]. However, in our study, the infection stage sample sizes for recent (*n* = 69) and chronic (*n* = 12) were smaller than acute (*n* = 265), which could make the comparisons less robust. In addition, we found significant differences between the median AUC values of primary HIV-1 isolates derived from CD4^+^ T cells or plasma (*p*-value = 0.007). Although it has been previously observed in our lab that the generation of primary HIV-1 isolates using patient plasma is more challenging than using CD4^+^ T cells, we currently have no explanation as to what could instigate these differences in the replication capacities. We intend to further explore that in the future. Furthermore, we compared the viral titers of the virus stocks to the RCs of the primary HIV-1 isolates. We did not observe a correlation between the viral titers of the virus stocks and the RC values, which was expected because all viruses were normalized to the same input amount of 200 TCID_50_/well.

Due to the numerous HIV-1 subtypes circulating the globe, it was important for our assay to be able to determine the replication capacities of HIV-1 subtype B, as well as HIV-1 non-B subtypes. We demonstrated that our assay was viable for 13 HIV-1 subtypes, including 7 circulating recombinant forms (CRFs). Furthermore, we compared the replication capacities of the primary HIV-1 isolates of the 13 different HIV-1 subtypes and exposed significant differences among the replication capacities between the HIV-1 subtypes (*p*-value < 0.0001). 

In summary, our high throughput parallel replication capacity assay can determine the replication capacities of extensive sample sizes of primary HIV-1 isolates in the same primary cells in one large experiment. Furthermore, it yields robust and reproducible results that can be exploited for data analysis.

## Figures and Tables

**Table 1:** Characteristics of primary HIV-1 isolates.

Primary HIV-1 Isolates (n)		346
HIV-1 stage of infection	Acute (n, %)	265 (76.6)
	Recent (n, %)	69 (19.9)
	Chronic (n, %)	12 (0.03)
Patient’s sample for generation of primary HIV-1 isolate	CD4 (n, %)	322 (93)
	Plasma (n, %)	24 (7)
Days in culture (average, (min, max))		20.4 (5, 48)
log TCID_50_/mL (average, [min, max])		5.28 (3.45, 8.16)
HIV-1 subtype	B (n, %)	256 (74.0)
	CRF01_AE (n, %)	28 (8.1)
	A (n, %)	18 (5.2)
	C (n, %)	11 (3.2)
	CRF02_AG (n, %)	10 (2.9)
	F (n, %)	7 (2.0)
	G (n, %)	4 (1.2)
	D (n, %)	3 (0.9)
	Other (n, %)	9 (2.5)

## Data Availability

The data presented in this study are available on request from the corresponding author. The data are not publicly available due to patient privacy.

## Supplementary Materials

The following are available online at https://www.mdpi.com/1999-4915/13/3/404/s1, Figure S1: Characterization of replication capacities of control viruses, Figure S2: Background controls maintain relatively stable over course of experiment, Figure S3: Replication kinetics of control viruses on every fourth plate of the high-throughput parallel infection assay.
