# Supplementary material for: A Novel High Throughput, Parallel Infection Assay for Determining the Replication Capacities of 346 Primary HIV-1 Isolates of the Zurich Primary HIV-1 Infection Study in Primary Cells
Source: Viruses. 2021 Mar 4;13(3):404. doi: 10.3390/v13030404 (PMC8000554; doi:10.3390/v13030404)
Supplement: Supplementary file 1 [file viruses-13-00404-s001.pdf]

Supplemental Material

Supplemental Figure 1:

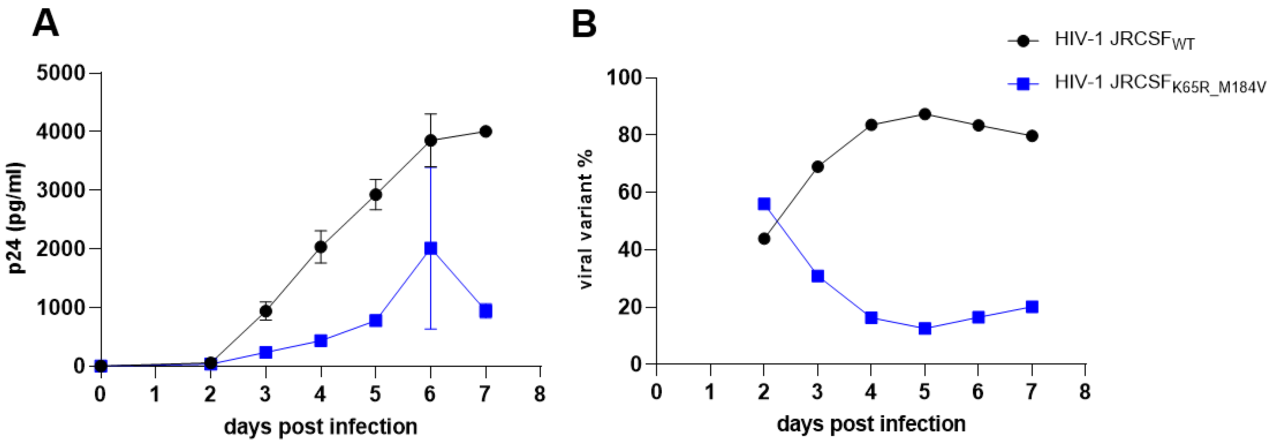

Supplementary figure 1. Characterization of replication capacities of control viruses. A) The graph compares the replication kinetics of HIV-1 JR-CSFWT (black dot) and HIV-1 JRCSFK65R_M184V (blue square) obtained from parallel infection. The x-axis represents the days post infection, and the y-axis represents the p24 antigen value in pg/ml (linear-scale). B) The graph depicts the dual-competition results between HIV-1 JR-CSFWT (black dot) and HIV-1 JRCSFK65R_M184V (blue square). The x-axis represents the days post infection, and the y-axis represents the percentage of HIV-1 JR-CSFWT and HIV-1 JRCSFK65R_M184V.

Supplemental Figure 2:

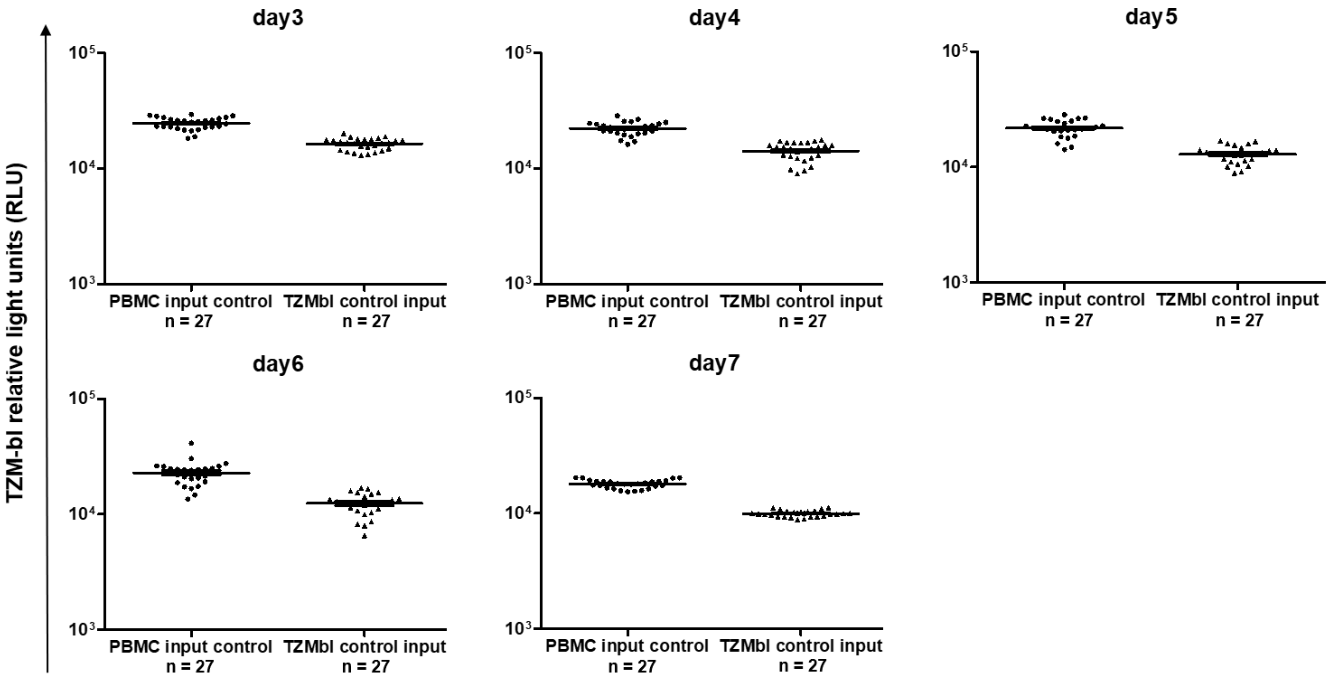

Supplementary figure 2. Background controls maintain relatively stable over course of experiment. These graphs depict the mean values (n = 8) of each control row on each plate for PBMC and TZM-bl controls from day 3 to day 7, from one experiment. The x-axes denote the PBMC and TZM-bl controls from 27 plates on each day post infection (3-7). The y-axes denote the average of each row from each plate (n = 27) of the TZM-bl luciferase expression measured in relative light units (RLU) for both controls.

Supplemental Figure 3:

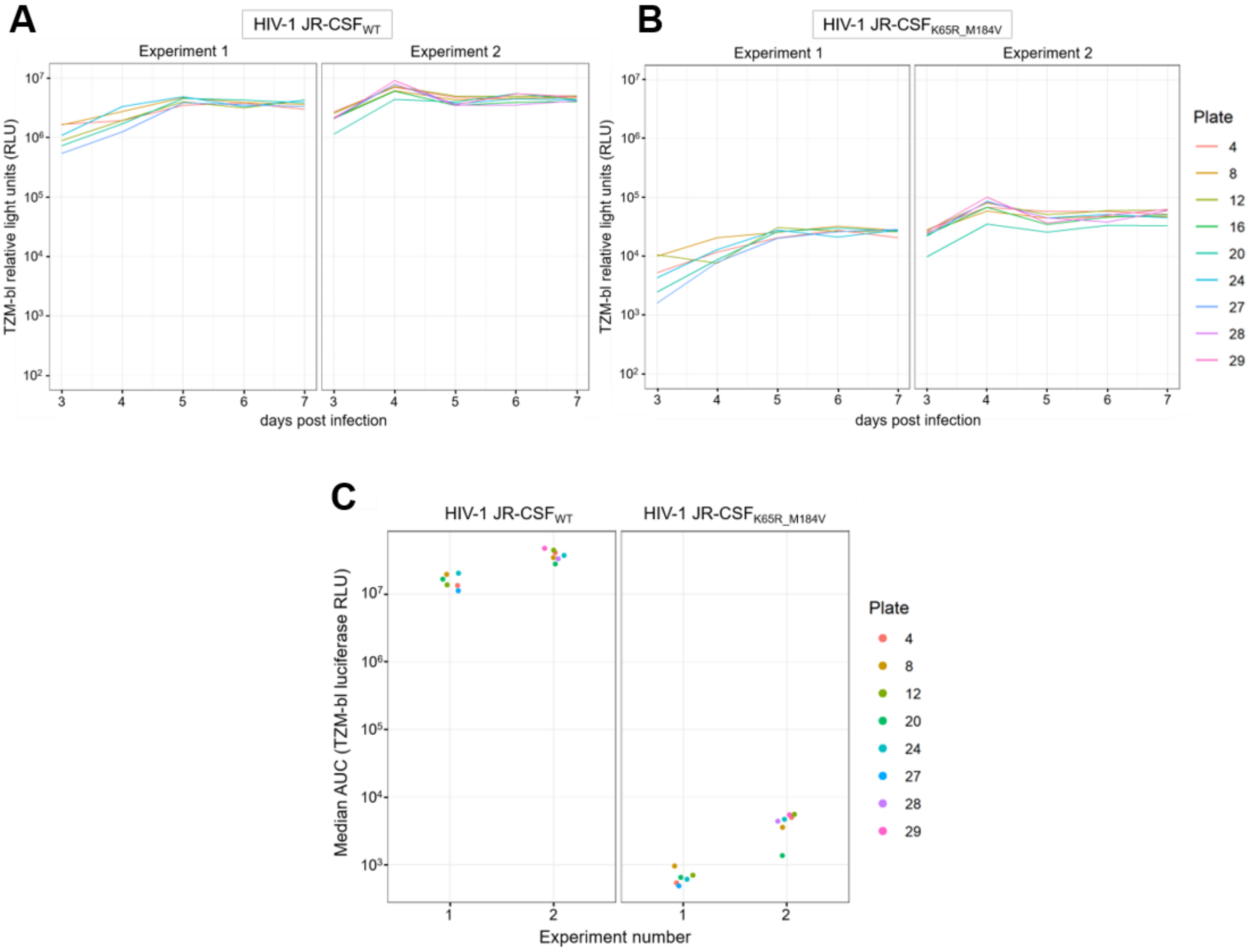

Supplementary figure 3. Replication kinetics of control viruses on every fourth plate of the high-throughput parallel infection assay. A-B) The graphs depict the replication kinetics for HIV-1 JR-CSFWT (A) and HIV-1 JR-CSFK65R_M184V (B) for two separate experiments. The x-axes represent the days post infection, and the y-axes denote the luciferase expression measured as relative light units (RLU). The different line colors represent the plate number that the control viruses were on. C) The jitter plots depict the median area under the curve (AUC) for the replication kinetics of the control viruses on every 4th plate. The x-axis represents which experiment the values were obtained, and the y-axis represents the median AUC measured by the TZM-bl luciferase relative light units.
